# Mixed Bacteriophage MS2-L2 VLPs Elicit Long-Lasting Protective Antibodies against HPV Pseudovirus 51

**DOI:** 10.3390/v13061113

**Published:** 2021-06-10

**Authors:** Rashi Yadav, Lukai Zhai, Nitesh K. Kunda, Pavan Muttil, Ebenezer Tumban

**Affiliations:** 1 Department of Biological Sciences, Michigan Technological University, Houghton, MI 49931, USA; rashiy@mtu.edu (R.Y.); Lukai.Zhai@vai.org (L.Z.); 2 Center for Cancer and Cell Biology, Van Andel Research Institute, Grand Rapids, MI 49503, USA; 3 Department of Pharmaceutical Sciences, College of Pharmacy, University of New Mexico, Albuquerque, NM 87131, USA; kundan@stjohns.edu (N.K.K.); PMuttil@salud.unm.edu (P.M.); 4 Department of Pharmaceutical Sciences, College of Pharmacy and Health Sciences, St. John’s University, Jamaica, NY 11439, USA; 5 School of Veterinary Medicine, Texas Tech University, Amarillo, TX 79106, USA

**Keywords:** human papillomaviruses, thermostable vaccines, protection, MS2 bacteriophage, virus-like particles, spray-freeze-drying, longevity

## Abstract

Three prophylactic vaccines are approved to protect against HPV infections. These vaccines are highly immunogenic. The most recent HPV vaccine, Gardasil-9, protects against HPV types associated with ~90% of cervical cancer (worldwide). Thus, ~10% of HPV-associated cancers are not protected by Gardasil-9. Although this is not a large percentage overall, the HPV types associated with 10% of cervical cancer not protected by the current vaccine are significantly important, especially in HIV/AIDS patients who are infected with multiple HPV types. To broaden the spectrum of protection against HPV infections, we developed mixed MS2-L2 VLPs (MS2-31L2/16L2 VLPs and MS2-consL2 (69-86) VLPs) in a previous study. Immunization with the VLPs neutralized/protected mice against infection with eleven high-risk HPV types associated with ~95% of cervical cancer and against one low-risk HPV type associated with ~36% of genital warts & up to 32% of recurrent respiratory papillomatosis. Here, we report that the mixed MS2-L2 VLPs can protect mice from three additional HPV types: HPV51, which is associated with ~0.8% of cervical cancer; HPV6, which is associated with up to 60% of genital warts; HPV5, which is associated with skin cancers in patients with epidermodysplasia verruciformis (EV). Overall, mixed MS2-L2 VLPs can protect against twelve HPV types associated with ~95.8% of cervical cancers and against two HPV types associated with ~90% of genital warts and >90% recurrent respiratory papillomatosis. Additionally, the VLPs protect against one of two HPV types associated with ~90% of HPV-associated skin cancers in patients with EV. More importantly, we observed that mixed MS2-L2 VLPs elicit protective antibodies that last over 9 months. Furthermore, a spray-freeze-dried formulation of the VLPs is stable, immunogenic, and protective at room temperature and 37 °C.

## 1. Introduction

Human papillomaviruses (HPVs) are non-enveloped, double-stranded DNA viruses [1,2]. There are more than 220 different types of HPVs [1,2]. Approximately 40 types are transmitted sexually through anogenital sex or oral sex [3,4,5]. HPVs are the most common sexually transmitted infections. Infection with high-risk types (HPV types 16, 18, 26, 31, 33, 35, 39, 45, 51–53, 56, 58, 59, 66, 68, 70, 73, and 82) at the anogenital region is associated with anogenital cancers (cervical, vaginal, vulval, penile, and anal cancers). In the oral region, infection is associated with head and neck cancers (oral squamous cell carcinomas, oropharyngeal squamous cell carcinomas, and laryngeal squamous cell carcinomas) [5,6,7,8]. On the other hand, infection of the anogenital region with low-risk HPV types (HPV types 6, 11, 40–44, 54, 61, 72, 81, etc.) is associated with genital warts [5,8]. HPV6 and 11 are associated with ~90% of genital warts. HPV5 and HPV8 are associated with ~90% of skin cancers in patients with a rare inherited cell-mediated immune skin disorder (epidermodysplasia verruciformis) [9].

Currently, three prophylactic vaccines (Gardasil-9, Cervarix, and Gardasil-4, discontinued in the U.S.) have been approved to protect against HPV infections. The vaccines are based on virus-like particles (VLPs) derived from the expression of the major capsid protein, L1 [10]. L1-based VLPs vaccines are highly immunogenic; however, they protect mostly against the HPV types included in the vaccines [11]. For example, Gardasil-9 (the most recent HPV vaccine) offers protection mostly against HPV types included in the vaccine. It protects against seven HPV types (16, 18, 31, 33, 45, 52, and 58) associated with approximately 90% of cervical cancers worldwide and against two HPV types (6 and 11) associated with approximately 90% of genital warts [8]. Thus, vaccinated individuals, especially HIV/AIDS patients (normally infected with multiple HPV types), can still be infected with HPV types not included in the vaccines [8]. Vaccinated individuals are advised to continue screening for HPV-associated cancers. There is, therefore, a need for an improved HPV vaccine: one that can protect against more HPV types.

The HPV capsid is made up of two capsid proteins: the major capsid protein (L1) and the minor capsid protein (L2). The capsid proteins assemble to form a virus particle or VLP as follows: first, the L1 protein forms pentamers, and the L2 protein is inserted (partially buried) at the vertex of each pentamer. Seventy-two copies of the pentamers then assemble to form a virus particle or VLP [12]. During HPV infection, the L1 protein makes contact with heparin sulfate proteoglycans on the basement membrane. This leads to a conformational change, and the L2 protein is transiently exposed to the surface, thus allowing the virus to bind to receptors on epithelial cells [11,13,14]. The L2 protein is highly conserved among different HPV types, unlike the L1 protein. Thus, candidate vaccines targeting the L2 protein are broadly protective. Studies including those from our group show that immunization with L2 peptides protects against diverse HPV types [15,16,17,18,19]. In our previous works [15,20], we demonstrated that immunization with a mixture of bacteriophage MS2 VLPs (MS2-31L2/16L2 VLPs and MS2-consL2 (69-86) VLPs [20]) offered protection against genital and oral infection with HPV pseudoviruses (PsVs) representing HPV11, 16, 18, 31, 33, 45, 53, 56, and 58 (at the genital region) and HPV16, 35, 39, 52, and 58 (in the oral region). Because low- and middle-income countries are significantly affected by HPV infection and because they have an underdeveloped cold-chain infrastructure for storing and distributing vaccines, we formulated mixed MS2 VLPs into a thermostable vaccine to address the cold-chain challenge. We demonstrated that mixed MS2-L2 VLPs could be spray-freeze-dried (SFD) into a thermostable product and that the SFD VLPs were immunogenic. In the current study, we assessed: (i) whether non-SFD-mixed MS2-L2 VLPs (MS2-31L2/16L2 VLPs and MS2-consL2 (69-86) VLPs) can protect against additional HPV types (HPV5, 6, and 51), (ii) whether SFD-mixed MS2-L2 VLPs are thermostable at room temperature for 6 months and at 37 °C for 2 months, and (iii) the longevity of protection (against HPV51) following immunization with the mixed MS2-L2 VLPs.

## 2. Materials and Methods

### 2.1. Production of MS2-L2 VLPs

**Figure 1:**
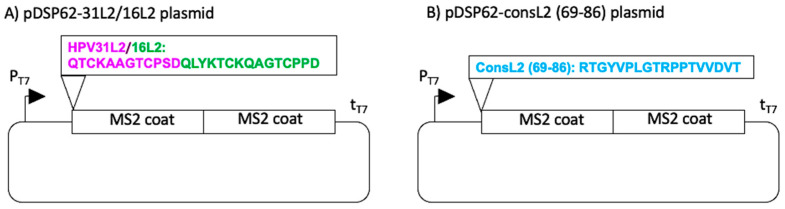
Plasmids with L2 sequences that were used for protein expression. 31L2/16L2 sequence (**A**) and consL2 (69-86) sequence (**B**) were inserted into plasmid by polymerase chain reaction in a previous study [15].

Plasmids encoding recombinant MS2-L2 proteins have previously been described (Figure 1) [15]. The plasmids encode proteins for: (i) MS2-31L2/16L2 VLPs (i.e., MS2 coat protein with the insertion of a concatemer of HPV L2 containing amino acids (aa) 20-31 from HPV31 L2 and aa 17-31 from HPV16 L2) and (ii) MS2-consL2 (69-86) (i.e., MS2 coat protein with an insertion of a consensus HPV L2 epitope, aa 69-86). C41 *Escherichia coli* bacteria were separately transformed by heat shock with the two recombinant plasmids (expressing MS2-31L2/16L2 VLPs and MS2-consL2 (69-86) VLPs). Cultures of transformed bacteria were grown at 37 °C until an optical density (OD)_600_ of 0.6, and the cultures were induced with 0.5 mM isopropyl β-D-1-thiogalactopyranoside for 3 h. The bacteria were pelleted and lysed with 0.2% lysozyme solution (for MS2-L2 consL2 (69-86)) or 10 mM borax solution (for MS2-31L2/16L2 VLPs). The VLPs were precipitated using 50% (*w/v*) ammonium sulfate and purified by gel filtration using Sepharose CL-4B columns.

### 2.2. Thermostability of SFD MS2-L2 VLPs

Spray-freeze-dried MS2 VLPs or mixed MS2-L2 VLPs powders (filled in capsules without adjuvants) have previously been described [20]. Briefly, the VLPs were formulated as follows: mixed MS2-L2 VLPs or control MS2 VLPs were added to a 3% *w/v* MTDL excipient solution, which contained mannitol (M, 75% *w/w*), trehalose (T, 7.5% *w/w*), dextran (D, 2.5% *w/w*), and L-leucine (L, 15% *w/w*). The VLPs were added at a concentration of 8% *w/w* to the MTDL excipients, and the VLPs-excipients were then spray-freeze-dried (SFD) [20]. SFD VLPs were loaded into capsules and stored in sealed bottles at room temperature for 6 months or at 37 °C for 2 months. Following these time points, the powders were reconstituted in 1X phosphate-buffered saline (PBS), and the integrity of VLPs was assessed by transmission electron microscopy (TEM) as follows: VLPs were adsorbed on carbon-coated glow-discharged copper grids for 2 min and were negatively stained with 2% uranyl acetate for 2 min. VLPs were visualized using an FEI 200 KV Titan Themis S-TEM (FEI now ThermoFisher, Waltham, MA, USA) and images were taken at 40,000X. The immunogenicity of the reconstituted VLPs was determined by immunizing mice as described below.

### 2.3. Immunization of Mice and Assessing Antibody Responses

Animal work was conducted following Michigan Technological University Institutional Animal Care and Use Committee guidelines (Protocol Number: L0264). Groups of 5 female BALB/c mice were immunized intramuscularly with 10 μg of mixed MS2-L2 VLPs (5 μg each of MS2-31L2/16L2 VLPs and MS2-consL2 (69-86)) in the presence of alum hydroxide (Alhydrogel, San Diego, CA, USA). 10 μg amounts of reconstituted SFD-mixed MS2-L2 VLPs stored at room temperature or 37 °C were also used to immunize mice in the presence of alum hydroxide. Control mice were immunized with 10 μg each of Gardasil-9, MS2 VLPs, and spray-freeze-dried MS2 VLPs (with alum). All immunizations were performed twice at two-week intervals. To assess the longevity of immune responses, 6 female BALB/c mice were immunized, thrice, with 10 μg of mixed MS2-L2 VLPs with alum at two-week intervals. The mice were immunized thrice because the concentration of each of the L2 VLP in the mixture of VLPs was only 5 μg. Whole blood was collected 2 weeks after the last immunizations (to assess antibody titers in sera) except from the groups of mice that were immunized with VLPs for longevity studies. In these groups of mice, sera were collected monthly. IgG antibody titers in sera were conducted by peptide enzyme-linked immunosorbent assay (ELISA) using the following peptides as target antigens: (i) 16L2 peptide, (ii) 31L2 peptide, and (iii) HPV consL2 (69-86) [21]. Briefly, 96-well plates were coated with 500 ng of peptide conjugated to streptavidin or biotin. The plates were blocked for 2 hours with 0.5% nonfat milk in 1X PBS. Sera from immunized mice were serially diluted (4-fold) and were added to the coated ELISA plates for 2 h. The plates were washed with 1X PBS, and horseradish peroxidase (HRP)-conjugated goat anti-mouse IgG antibodies (1:5000 dilution) were added for 1 hours. The wells were washed and developed with TMB solution (3, 3′, 5, 5′-tetramethylbenzidine). The reaction was stopped with 1 M hydrochloric acid. Absorbance was measured at OD_450nm_ using the Synergy LX multimode reader (BioTek, Winooski, VT, USA). Antibody titers were determined by considering the reciprocal of the highest sera dilutions at which the reactivity of experimental sera was greater than 2-fold compared to control sera at the same dilution.

### 2.4. Genital Infection

HPV pseudoviruses (PsVs) representing HPVs 5, 6, and 51 were made and purified by cesium chloride gradient ultracentrifugation as described previously [22,23]. In brief, PsVs were made by co-transfecting 293TT cells with equal concentrations of pSheLL plasmid and pClucf plasmid. pSheLL plasmid expresses the capsid proteins of the HPV type for the pseudovirus of interest, while pClucf plasmid expresses two reported proteins (luciferase and green fluorescence protein). Two days after transfections, the cells were harvested and lysed, and the lysates were incubated overnight at 37 °C to allow the PsVs to mature. The lysates were spun down, and the supernatant was put on a discontinuous cesium chloride gradient (1.25 and 1.4 g/mL, *w/v*). The gradient was spun for 17 h (4 °C) at 20,000 rpm, and a band corresponding to HPV PsVs was collected. HPV PsVs contain pClucf plasmids, which can be used to assess infectivity based on the expression of the reporter genes (luciferase and green fluorescence protein) in infected cells. Infection studies were conducted as previously described [22,24,25]. Concisely, immunized mice were subcutaneously injected with 3 mg of Depo-Provera. Five days after Depo-Provera treatment, the genitals of mice were treated with 4% nonoxynol-9, and the mice were vaginally challenged (5 h later) with ~6.4 × 10^6^ infectious units (IUs) of PsVs as described previously. Forty-eight hours after PsV infection, mice were genitally instilled with 0.4 mg of luciferin (a substrate for luciferin). Mice were imaged using IVIS Spectrum at one-minute exposure to capture the intensity of luciferase expression (bioluminescence, which is indicative of the level of infection). Average radiance (*p*/cm^2^/sr) was determined by drawing equally sized regions of interest surrounding the site of PsV administration.

### 2.5. Statistical Analysis

Statistical analysis of ELISAs and HPV PsV challenge studies were performed using an unpaired two-tailed *t*-test and an unpaired one-tailed *t*-test, respectively.

## 3. Results

### 3.1. Mixed MS2-L2 VLPs Protect against HPV PsVs 5, 6, and 51

**Figure 2:**
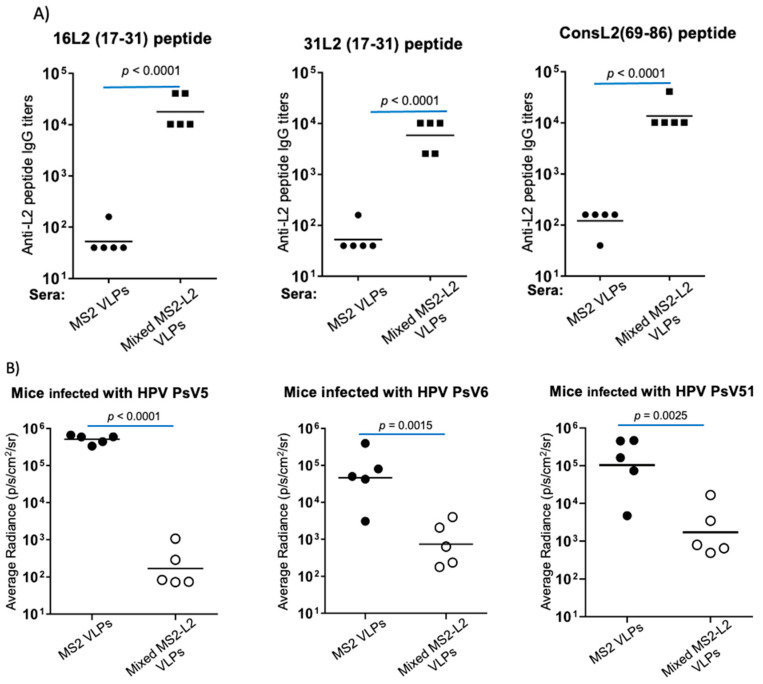
Immunogenicity of mixed MS2-L2 VLPs with alum. Mice were immunized intramuscularly, twice, with 10 μg of mixed MS2-L2 VLPs or control MS2 VLPs with alum at two-week intervals. (**A**) Sera were collected 2 weeks after the last immunization, and anti-L2 peptide IgG titers in sera were determined by end-point dilution ELISA using the following peptides as target antigens: HPV16 L2 peptide, HPV31 L2, and HPV consL2 (69-86). (**B**) The mice in A above were genitally challenged with 6.4 × 10^6^ IU of: HPV PsV5, HPV PsV6, and HPV PsV51. The average radiance (*p*/s/cm^2^) of luciferase expression was determined using Living Image software version 4.5.5. Each datum represents the average radiance of an individual mouse, and the lines represent the geometric mean for each group of mice. The *p*-values were determined by an unpaired two-tailed *t*-test for 2A and an unpaired one-tailed *t*-test for 2B.

We previously showed that non-SFD-mixed MS2-L2 VLPs are immunogenic [20]. However, to confirm the immunogenicity of the VLPs prior to the protection studies, we performed an ELISA using sera from new groups of immunized mice (to be infected). Figure 2A shows that the antibody titers (>10^3^) were similar to those from our previous study [20]. High-titer antibodies were elicited against all three L2 peptides tested (16L2 aa 17-31, 31L2 aa 17-31, and consensus L2 69-86). Given the immune responses, we decided to assess whether immunized mice would be protected from genital infection with HPV PsVs 5, 6, and 51. In the genital region, if anti-HPV L2 antibodies were present (due to vaccination), they would block entry of the PsVs into cells in the region, and thus, pClucf would not be delivered into the nuclei for the reporter genes (e.g., luciferase) to be expressed. Luciferase expression is a read-out for infection. However, if anti-L2 antibodies were absent, the PsVs would enter cells in the genital region, leading to the expression of reporter genes in the nuclei. PsV infection is not productive (i.e., the PsVs do not replicate, and thus, they cannot spread from cell to cell). As shown in Figure 2B, mice immunized with the non-SFD-mixed MS2-L2 VLPs were protected against the three HPV PsVs. Mice immunized with non-SFD-mixed MS2-L2 VLPs had low average radiances (luciferase expression) compared to control mice. Protection against HPV PsV5 was better compared to protection against HPV PsVs 6 and 51.

### 3.2. Spray-Freeze-Dried Mixed MS2-L2 VLPs Are Thermostable at Room Temperature for 6 Months and at 37 °C for 2 Months

**Figure 3:**
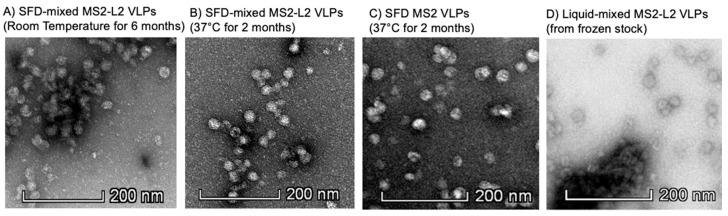
Transmission electron microscopy (TEM) images of spray-freeze-dried (SFD) VLPs and liquid VLPs. Spray-freeze-dried VLPs were reconstituted in PBS and analyzed using TEM. (**A**) TEM of SFD-mixed MS2-L2 VLPs stored at room temperature for 6 months. (**B**) TEM of SFD-mixed MS2-L2 VLPs stored at 37 °C for 2 months. (**C**) TEM of spray-freeze-dried control MS2 VLPs stored at 37 °C for 2 months. (**D**) Liquid-mixed MS2-L2 VLPs (from frozen stock). Images were taken at 40,000× magnification.

**Figure 4:**
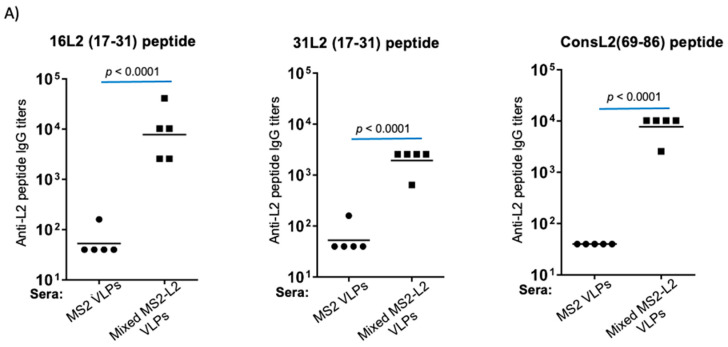

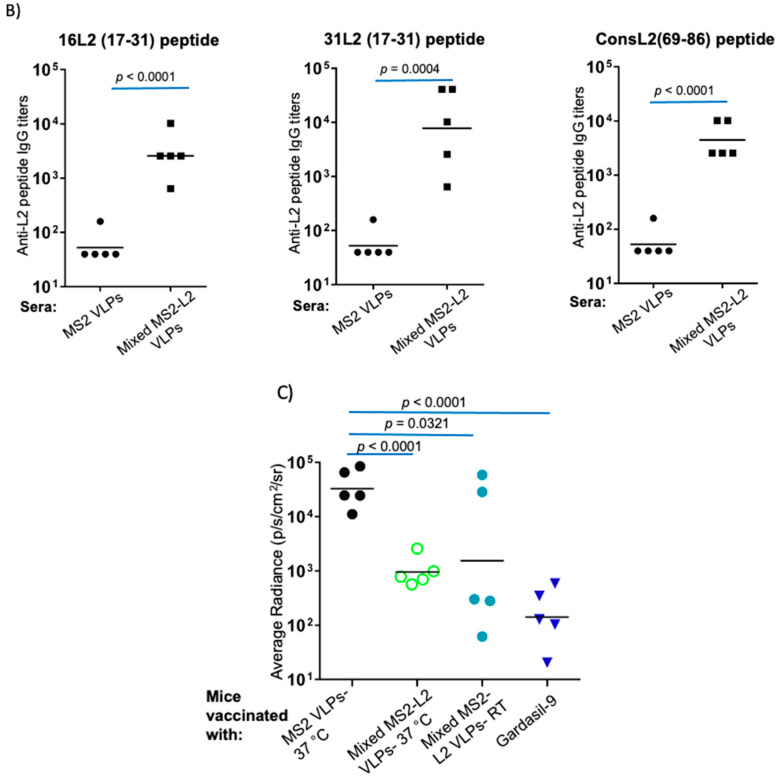
Immunogenicity of SFD-mixed MS2-L2 VLPs and protection from genital infection. Mice were immunized, twice, intramuscularly with 10 μg of spray-freeze-dried VLPs with alum at two-week intervals. Sera were collected 2 weeks after the last immunization. Anti-L2 peptide IgG titers in sera were determined by end-point dilution ELISA using 16L2 peptide, 31L2 peptide, and consL2 (69-86) peptide as target antigens. The sera were derived from mice immunized with: (**A**) SFD-mixed MS2-L2 VLPs and SFD MS2 VLPs stored at room temperature for 6 months and (**B**) SFD MS2 VLPs and SFD-mixed MS2-L2 VLPs stored at 37 °C for 2 months. Immunized mice in 3A and 3B, including a positive control group, immunized with 10 μg of Gardasil-9, were genitally challenged with 6.4 × 10^6^ infectious units of HPV PsV6 (**C**). Each datum represents the average radiance of an individual mouse, and the lines represent the geometric mean for each group of mice. The *p*-values were determined by an unpaired two-tailed *t*-test for Figure 4A,B and an unpaired one-tailed *t*-test for Figure 4C.

To assess at what temperature and for how long the SFD-mixed MS2-L2 VLPs can be stored without refrigeration, the SFD VLPs were stored at room temperature for 6 months and at 37 °C for 2 months, and their integrities were assessed using TEM. As shown in Figure 3, spray-freeze-dried VLPs stored at either temperature did not disintegrate. To assess whether the SFD VLPs stored at room temperature or 37 °C were immunogenic, reconstituted spray-freeze-dried VLPs were used to immunize mice. SFD-mixed MS2-L2 VLPs stored at room temperature for 6 months elicited high-titer anti-IgG peptide antibodies against HPV16 L2 and consensus L2 (69-86) peptides, while suboptimal antibodies (in comparison to non-SFD VLPs in Figure 2A) were detected against the HPV31 L2 peptide (Figure 4A). SFD-mixed MS2-L2 VLPs stored at 37 °C for 2 months were also immunogenic (Figure 4B); however, their overall immune responses against two L2 peptides (HPV16 L2 and consL2 (69-86)) were lower compared to the VLPs that were stored at room temperature or non-SFD in Figure 2A. To assess if the elicited immune response (at least >10^3^) could offer protection against HPV pseudovirus infection, immunized mice were genitally infected with HPV PsV6. As shown in Figure 4C, SFD-mixed MS2-L2 VLPs offered protection. Three out of five mice from the group that was immunized with SFD-mixed MS2-L2 VLPs stored at room temperature (for 6 months) offered protection (similar to Gardasil-9) against HPV PsV6 compared to SFD-mixed MS2-L2 VLPs stored at 37 °C for 2 months. Two mice from the group that was immunized with SFD-mixed MS2-L2 VLPs stored at room temperature were not protected at all. Overall, Gardasil-9-immunized mice (refrigerated) offered the best protection compared to the mice immunized with spray-freeze-dried VLPs stored at the above temperatures and time frames.

### 3.3. Mixed MS2-L2 VLPs Elicit Long-Lasting Antibody Response against PsV51

**Figure 5:**
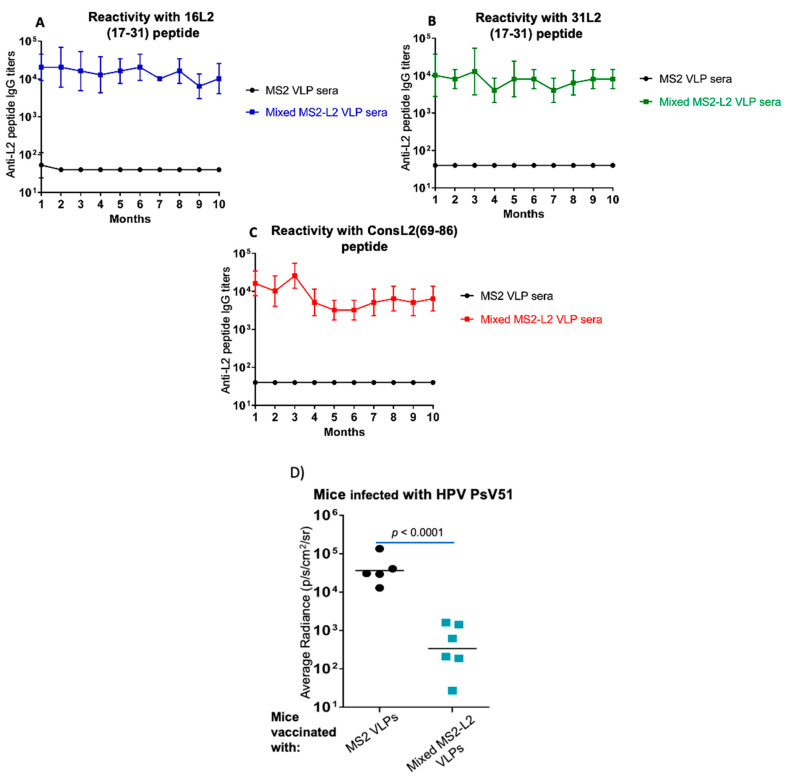
Longevity of mixed MS2-L2 VLP antibodies. Mice were immunized, thrice, intramuscularly with 10 μg (each) of mixed MS2-L2 VLPs with alum at two-week intervals. Sera were collected monthly (for 10 months) after the last immunization. Anti-L2 peptide IgG titers in sera were determined by end-point dilution ELISA using the following peptides as target antigens: (**A**) HPV16 L2, (**B**) HPV31 L2, and (**C**) HPV consL2 (69-86). (**D**) Immunized mice were challenged after 10 months with 6.4 × 10^6^ infectious units of HPV PsV51. Each datum represents the average radiance of an individual mouse, and the lines represent the geometric mean for each group of mice. The *p*-value was determined by an unpaired one-tailed *t*-test for Figure 5D.

To assess how long the immune responses elicited by mixed MS2-L2 VLPs would last, mice were immunized with non-SFD-mixed MS2-L2 VLPs, and antibody titers were monitored monthly for 10 months. As shown in Figure 5A–C, antibody titers stayed fairly constant against HPV16 L2 (aa 17-31) and HPV31 L2 (aa 17-31) peptides but minimally declined against consensus L2 (69-86) peptide. More importantly, the mice were protected from infection against HPV PsV51 10 months after immunization (Figure 5D).

## 4. Discussion

Three prophylactic vaccines are approved to protect against HPV infections. These vaccines are very immunogenic. However, they protect mostly against HPV types included in the vaccines. For example, Gardasil-9, which has the most HPV VLP types (HPV 6, 11,16, 18, 31, 33, 45, 52, and 58) included in the vaccines, protects against HPVs associated with ~90% of cervical cancer, 80–85% of HPV-associated vaginal cancer, 85–90% of HPV-associated vulvar cancer, 90–95% of HPV-associated anal cancer, ~90% of genital warts, and ~86% of HPV-associated penile cancer [8]. It is also anticipated that the vaccine will protect against HPV types associated with ~93% of HPV-associated head and neck cancers. The vaccine also protects against approximately 90% of genital warts [8]. Given the fact that the L1-based vaccines do not protect against all HPV types associated with cancers and the fact that most people infected with HPV may be infected with more than one HPV type (especially HIV/AIDS patients), an alternative target for a broadly protective HPV vaccine has been under exploration for many years. For example, the L2 protein has had a lot of attention (within the last two decades) as a target antigen to develop a next-generation vaccine against HPVs [26,27,28,29]. Immunization with peptides derived from the N-terminus of L2 elicits antibodies that cross-protect against diverse HPV types [16]. In previous works, we demonstrated that immunization with a mixture of two MS2-L2 VLPs (MS2-31L2/16L2 VLPs and MS2-consL2 (69-86) VLPs) offers protection from genital infection with eight high-risk HPV types (HPV PsV16, 18, 31, 33, 45, 53, 56, and 58) and one low-risk HPV type (HPV PsV11). We also demonstrated that the candidate vaccine can offer protection from oral infection with five high-risk types (HPV PsV16, 35, 39, 52, and 58) [15,20]. Here, we show that the candidate vaccine can protect against three additional HPV types: non-SFD-mixed MS2-L2 VLPs also protect against HPV PsV51 (a high-risk HPV type), HPV PsV6 (a low-risk type associated with genital/oral infection), and HPV PsV5. HPV5 is a low-risk HPV, and it is one of two low-risk HPVs (including HPV8) associated with ~90% of HPV-associated skin cancer in patients suffering from epidermodysplasia verruciformis. Protection against HPV PsV5 was better compared to protection against HPV PsV6 and PsV51. We believe this was because part of HPV16 peptide (amino acid 20-31), which was displayed on one of the VLPs is 100% identical to that of HPV5. This amino acid sequence is only 81–90% identical to those of HPV6 and HPV51. These results are consistent with the reactivity of sera (from mice immunized with HPV16 L2 peptide 17-31) with peptides from HPV5 L2 and HPV6 L2 in our previous study [21].

Current HPV vaccines require cold-chain storage and may not be suitable for developing countries with economic challenges such as expenses to provide continuous refrigeration during transportation and storage. Exposure to aberrant temperature can decrease the efficacy of vaccines (in general) [30]. For example, we have shown that liquid MS2-L2 VLPs disintegrate after storage at room temperature for one month [31]. Hence, a thermostable vaccine will be a better solution to achieve widespread immunization for low- and middle-income countries with less-developed refrigeration facilities. Fortunately, in our previous study, we showed that VLPs are thermostable after spray-freeze-drying. We observed that SFD-mixed MS2-L2 VLPs could be stored at room temperature for 2 months without affecting immunogenicity and protection [20]. Here, we demonstrated that the VLPs could be stored for an additional 4 months (a total of 6 months) at room temperature or 37 °C for 2 months without a significant loss in the integrity of the VLPs. Sera from SFD VLPs stored at room temperature (6 months) had slightly higher reactivity against all HPV L2 peptides (except HPV 31L2) compared to sera from the same VLPs stored at 37 °C (2 months). Although antibody titers in sera from mice immunized with the VLPs stored at room temperature were slightly lower against HPV 31L2 peptide compared to those from VLPs stored at 37 °C, this difference was not significant (*p* = 0.1434) (Figure 4). Three out of five mice that were immunized with the SFD-mixed MS2-L2 VLPs stored at room temperature offered protection against PsV6 that was similar to that of Gardasil-9 but better than those in mice immunized with SFD VLPs stored at 37 °C. However, two mice in the group of SFD-mixed MS2-L2 VLPs stored at room temperature were not protected at all (Figure 4C). We are not sure why these two mice were not protected. Systemic immunizations elicit protective immunity at mucosal sites; however, they can also be incapable of eliciting protective mucosal immune responses [32]. We speculate that the two mice might not have had high-titer protective antibodies in the genital region (where the pseudovirus was instilled) despite high-titers in the sera.

In summary, mixed MS2-L2 VLPs are an ideal candidate HPV vaccine that should be explored further. They are highly immunogenic. Non-SFD-mixed MS2-L2 VLPs can protect against HPV PsVs5, 6, and 51. This is in addition to protection against HPV PsV11, 16, 18, 31, 33, 35, 39, 45, 53, 56, 52, and 58) in our previous studies [15,20]. Thus, mixed MS2-L2 VLPs have the potential to protect against twelve high-risk HPV types. These HPV types are associated with ~95.8% of cervical cancer [8] and ~99.5% of HPV-associated head and neck cancers [33,34,35,36]. Additionally, the mixed MS2-L2 VLPs have the potential to protect against low-risk HPV types (HPV6 and HPV11) associated with ~90% of genital warts and >90% recurrent respiratory papillomatosis. Estimates are based on the contribution of HPV6 and HPV11 in genital warts [37,38] and recurrent respiratory papillomatosis [39,40]. Moreover, immune responses elicited by non-SFD-mixed MS2-L2 VLPs lasted up to 10 months (tested so far) and were protective against HPV PsV51. Given the fact SFD-mixed MS2-L2 VLPs stored at room temperature for 6 months and at 37 °C for 2 months offered suboptimal protection from HPV PsV6, we recommend that the spray-freeze-dried VLPs should only be stored at room temperature for 2 months. SFD-mixed MS2-L2 VLPs stored at room temperature for 2 months offered complete protection from infection in our previous study [20]. Thus, they can be reconstituted on-site just prior to immunization. Such a candidate vaccine would be applicable in low and middle-income countries that do not have a robust cold-chain infrastructure for storing and distributing vaccines. The candidate vaccine would also be applicable in communities with HIV/AIDS patients, who may be susceptible to infections with multiple cancer-causing HPV types. Future studies are required to assess the longevity of immune responses to the SFD-mixed MS2-L2 VLPs and the immunogenicity of the VLPs in larger animal models such as rabbits or guinea pigs. MS2-16L2 VLPs are immunogenic in rabbits. Sera from rabbits immunized with MS2-16L2 VLPs neutralize HPV PsV16 (unpublished data); thus, it is worth exploring whether the immunization of rabbits with mixed MS2-L2 VLPs will elicit a broad response compared to immunization with only MS2-16L2 VLPs.

## Data Availability

Not Applicable.
